# Monitoring of Cleaning Treatments for Paper Heritage with Raman Spectroscopy Mapping

**DOI:** 10.3390/molecules29010015

**Published:** 2023-12-19

**Authors:** Sabina Botti, Francesca Bonfigli, Luca Mezi, Francesco Flora

**Affiliations:** 1 Photonics Micro and Nanostructures Laboratory, Physical Technologies for Safety and Health Division, Fusion and Technologies for Nuclear Safety Department, Italian National Agency for New Technologies, Energy and Sustainable Economic Development (ENEA), Via E. Fermi 45, 00044 Frascati, Italy; francesca.bonfigli@enea.it; 2 Plasma Applications and Interdisciplinary Experiments Laboratory, Plasma Studies Division, Fusion and Technologies for Nuclear Safety Department, Italian National Agency for New Technologies, Energy and Sustainable Economic Development (ENEA), Via E. Fermi 45, 00044 Frascati, Italy; luca.mezi@enea.it (L.M.); francesco.flora@enea.it (F.F.)

**Keywords:** Raman spectroscopy, cultural heritage, paper aging diagnostics, Raman spectral imaging, paper cleaning treatment

## Abstract

In the field of book heritage, it is important to develop cleaning/disinfecting treatments that can slow down the degradation of paper to prevent evident and irreversible damage. The objectives of the cleaning treatments are to remove external contaminants and oxidation and decomposition products of the paper, but these processes must not modify the unique characteristics of the book heritage resulting in irreversible changes in the structure of the paper. Recently, several innovative cleaning treatments were developed with the aim of being minimally invasive; however, to assess the effect of these treatments on paper, it is necessary to use a diagnostic non-destructive, rapid, and affordable process. In previous work, we used surface scanning Raman spectroscopy to develop a diagnostic protocol able to follow the aging processes of the paper, discriminating between hydrolysis and oxidation. In this paper, we applied this protocol to study the action of different types of treatments (hydrogel and EUV irradiation), evaluating both their effectiveness and impact on paper parameters. The results reported here demonstrate that the developed in operando diagnostic procedure can follow the changes in the paper structure comparing them to the variability due to the intrinsic inhomogeneity of paper, without sample contact in a rapid and effective way.

## 1. Introduction

Over the centuries, paper has made a great contribution to civilization. Ancient and old documents hold our history, therefore the development of suitable scientific approaches for the conservation and restoration of library heritage is attracting a growing interest. The cellulose is the main constituent of paper and it is a stable material, but, over time, it undergoes a natural degradation due to hydrolytic and oxidative chemical reactions. The hydrolysis of the cellulose chain can develop in both an acidic and basic environment, causing a breaking of the glucosidic bond between monomers with consequent depolymerization of the material. Oxidation-reduction reactions induce the attachment of functional groups to the cellulose backbone [1,2,3]. The level of degradation depends on: (a) the intrinsic characteristics of the paper such as, for example, the type of raw materials used for production, the manufacturing methods, the possible presence of added substances, etc.; (b) the nature of the materials used on it, for example, inks, pigments, binders, etc.; (c) the storage conditions: possible presence of pathogenic organisms, air pollutants, exposure to light, incompatible thermo-hygrometric parameters, etc.

Monitoring the conservation state of paper in parallel with the implementation of minimally invasive treatments for cleaning and restoration is the only way to avoid losing, perhaps forever, assets of inestimable value. To recognize the functional groups that are formed during aging is of prime importance for planning book heritage conservation, and although not quantitative, the spectroscopic diagnostics that do not come in contact with the sample and that are non-destructive, have gained much interest and are widely applied [4,5,6,7,8,9,10,11]. In particular, Raman spectroscopy is not affected by the water signal as opposed to infrared spectroscopy, in which the OH bending falls in the same region of C=C and C=O stretching. Raman spectra of hydrolyzed papers do not show additional peaks compared to modern papers, whereas oxidation, causing modification in the cellulose structure, produces the appearance of new peaks around 1580 cm^−1^, in the 1640–1740 cm^−1^ region and above 2000 cm^−1^ [9,12,13]. Starting from Raman spectra features as intensity and area of fingerprint peaks, it is possible to follow the aging processes of the paper, discriminating between hydrolysis and oxidation [12].

To slow down paper degradation and restore book heritage quality as much as possible, several cleaning treatments were developed [14]. The objectives of the cleaning treatments are to remove external contaminants and oxidation and decomposition products of the paper, but these processes must not modify the unique characteristics of the artifacts, resulting in irreversible changes in the structure of the paper. Therefore, to assess the best cleaning treatment parameters, Raman spectroscopy can be considered a suitable tool to evaluate the paper surface state before and after the treatment.

However, the paper surface is an inhomogeneous system and to perform this evaluation using the characteristics of a few Raman spectra is a cumbersome task. To better highlight the paper state variation, we acquired Raman maps before and after treatment in the same area. Our procedure consists of choosing a region of interest of the sample under examination, defining a grid of points, and acquiring a Raman spectrum for each point of the pre-set grid. We defined aging markers to be used as contrast parameters to build spectral maps co-localized with the image of the sample displayed under the optical microscope. From a single set of spectra, it is possible to obtain all the spectral maps relating to the different markers, that give information on oxidation, hydrolysis advancement, and crystallinity, describing the conservation state of paper and its characteristics.

In this work, we report the results obtained by applying this technique to different paper samples before and after two different cleaning treatments to assess their suitability and effectiveness and, most importantly, to characterize the possible modifications induced by the treatment in the structure of the paper. Overall, our measurements demonstrate that the developed procedure is a valuable tool for testing new and innovative treatments.

## 2. Results

In this section, we report the results obtained by applying the diagnostic protocol that we developed according to the characterization of three different standards of paper samples and to the in operando analysis of hydrogel and EUV irradiation-based cleaning treatment.

### 2.1. Raman Spectral Imaging of Standard Papers

**Figure 1:**
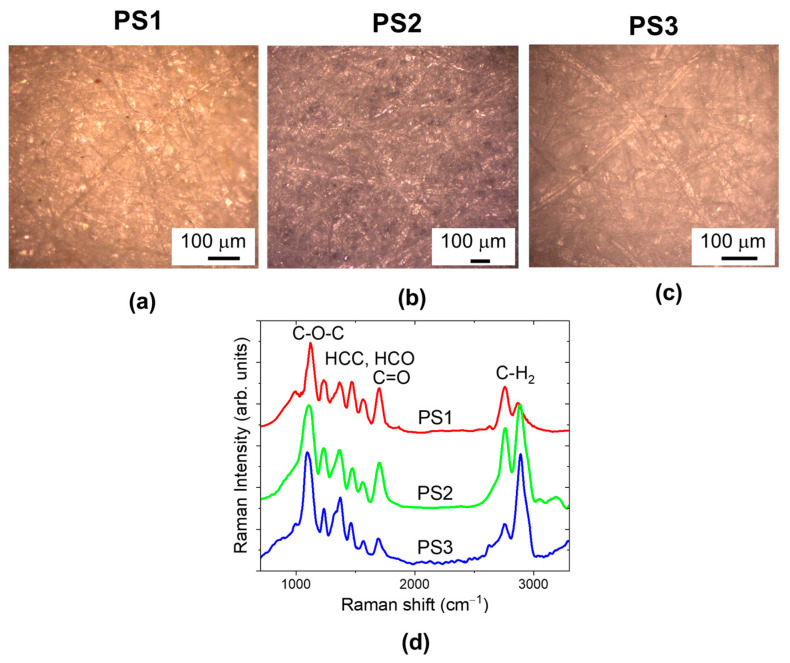
Optical images detected in white light of different paper samples: Antaimoro (PS1) (**a**); Funori (PS2) (**b**); Whatman (PS3) (**c**). (**d**) Representative Raman spectra, extracted from 2D (280 µm × 160 µm) Raman maps (448 spectra) detected for each paper samples.

Figure 1a–c show white light optical microscope images of different paper samples: Antaimoro (PS1), Funori (PS2), Whatman (PS3). The cellulose fibers were taken from the avoha semi-aquatic plant, red algae, and cotton, respectively.

For each paper, we acquired a set of Raman spectra over an area of 280 µm × 160 µm with a step size in x and y direction of 10 µm. Representative Raman spectra are reported in Figure 1d. All the observed peaks can be attributed to the main component of paper, i.e., cellulose. In previous assessments of cellulose, it was observed that only in the region below 1500 cm^−1^ are the modes simple group motions. The rest of the modes are delocalized motion involving more than one group or site in the molecule [15].

The C-O-C motions are observed in the 1180 cm^−1^ region, with a negligible contribution of HCC-HCO bending, that gives rise to the bands in the 1270–1500 cm^−1^ region. The bands at 1580 and 1740 cm^−1^ are due to C=O stretching. The CH_2_ group exhibits both symmetric and asymmetric stretching bands around 2890 cm^−1^.

According to data from the literature, we defined aging markers based only on the spectra parameters that are sensitive to the polymerization, oxidation, and crystallinity degree [12]. These markers were evaluated for each spectrum.

**Figure 2:**
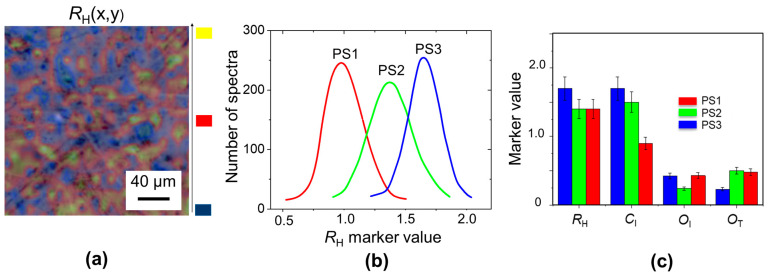
(**a**) Raman map of *R*_H_ marker (degree of polymerization of the cellulose) of PS3 paper sample, overlapped with its optical image; (**b**) *R*_H_ distributions for PS1, PS2, PS3 paper samples; (**c**) Peak values for *R*_H_, *C*_I_, *O*_I_, *O*_T_ markers for each paper samples.

Figure 2a shows, for the case of the PS3 paper sample, the Raman map of *R*_H_ marker, the value that is given by the ratio of the band intensity of the vibrations of C-O-C bond between monomers and of the C-H bond in the glucose monomer, following this formula:

*R*_H_ = I_C-O-C_/I_C-H_ = I_1100_ cm^−1^/I_1380_ cm^−1^ (1)

where I_Rs_ cm^−1^ is the intensity of the peak at Rs cm^−1^.

Given that the more C-O-C bonds there are, the longer the cellulose chain, the value of this marker is proportional to the degree of polymerization of the cellulose chain. Due to aging, the advancement of the hydrolysis process causes a diminution of the *R*_H_ parameter, from this parameter, the age of ancient paper was estimated [6].

In the color code used in these maps, the increasing intensity is displayed in the blue-red-yellow gradation, evidencing that the region with higher *R*_H_ is near the crossing of longer fibers. For each paper sample, a distribution of *R*_H_ values is calculated, as reported in Figure 2b. The distribution width ranges from 10 to 20% of peak value, as expected for an inhomogeneous system such as paper. The *R*_H_ peak values follow the degree of polymerization of the cellulose fibers from which paper samples are assembled. In fact, the highest value is that of the PS3 Whatman paper sample, which is derived from cotton fibers.

This procedure can be repeated by obtaining the maps, distribution, and peak value for the other markers:

*O*_I_ = A_1640–1850_ cm^−1^/A_1500–1600_ cm^−1^ (2)

*O*_T_ = A_1500–2800_ cm^−1^ /A_700–3000_ cm^−1^ (3)

*C*_I_= I_2890_ cm^−1^ /I_1380_ cm^−1^ (4)

where A_Rs1–Rs2_ cm^−1^ represents the area of the peaks in the Raman shift region between Rs1 and Rs2 cm^−1^.

The two parameters *O*_I_ and *O*_T_ have different meanings: the first one indicates that the oxidation process is advanced because the C=O bonds belong to the ultimate oxidation products, whereas the *O*_T_ parameter is proportional to the concentration of all oxidized functional groups linked to the cellulose backbone [12,13,14,15,16]. The index *C*_I_ [12] of cellulose crystallinity describes the decrease in CH_2_ peak intensity related to the reduction in the content of ordered (crystalline) components of cellulose. This marker decreases as a consequence of the shortening of cellulose chain length in paper aging treatment ([12], and references cited in [12]).

For a close comparison between the different paper standards, the obtained peak values for each marker are reported in Figure 2c. The paper samples with larger values of *R*_H_ and *C*_I_ parameters show a smaller value of the oxidation parameter *O*_T._ This is due to the fact that more ordered and longer cellulose fibers are also more resistant to oxidation attacks and functional group linkages. However, the *O*_I_ index is large, indicating that in the PS1 paper sample, there is a large content of C=O bonds, due to a more advanced oxidation stage.

**Figure 3:**
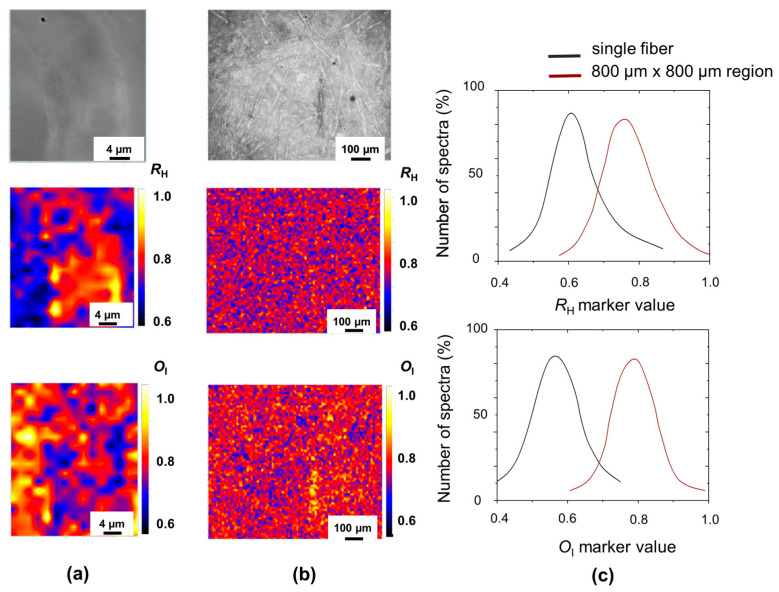
Upper (**a**,**b**): optical images in white light of XIX paper (from O. Tedone, Note sul moto di un fluido, Pisa, 1893) detected at two different magnifications: 100× (**a**), 5× (**b**). In the middle of (**a**,**b**): *R*_H_ maps of the same paper at the two different magnifications. Bottom (**a**,**b**): *O*_I_ maps of paper “Nota Tedone 1893” at the two different magnifications. (**c**) *R*_H_ and *O*_I_ distributions correspond to a higher magnification area (single fiber) and to a lower magnification area (800 µm × 800 µm).

Once we have defined the correct contrast parameters, it is possible to describe degradation patterns with different length scales, discriminating between more or less degraded areas on the same page. Simply by changing the microscope objective, it is possible to switch from the analysis of a single fiber or of a large area of paper sample, as reported in Figure 3 for a non-printed region of a XIX century book (O. Tedone, Note sul moto di un fluido, Pisa, 1893).

If we compare the correlated maps of *R*_H_ and *O*_I_ markers for the single fiber in Figure 3a, we observe that, as discussed before, the hydrolysis and oxidation processes proceed more slowly on an ordered structure, such as the fiber wall where we measured the highest *R*_H_ values, and the lowest *O*_I_ values. By increasing the sampled area, the corresponding marker distribution of *R*_H_ and *O*_I_ index moves to a larger peak value, see Figure 3c, as longer and more oxidized fibers were included (e.g., yellow fiber in the *O*_I_ maps reported in Figure 3b).

Then, it is a good practice for the comparison of different books to analyze areas of equal size and in the same position in the book: for example the bottom right edge of the first chapter page or of the index.

### 2.2. Application of Raman Spectroscopy with Mapping to Paper Cleaning Treatment Evaluation

In the field of book heritage, it is of prime importance to find a method that can prevent and slow the paper damage due to aging. The definition of a cleaning method will make it possible to preserve artworks, rare book editions, and documents.

The objectives of the cleaning treatment are to remove external pollutants, possibly present on the paper surface contaminants, and to remove oxidation and decomposition products of the paper, preserving the unique characteristics of the archival asset without resulting in irreversible changes in the structure of the paper. In regard to this, several innovative cleaning treatments were developed to be minimally invasive, and a diagnostic suitable for evaluating how much the treatment removes the contaminant stain and how much the paper characteristics are possibly affected by the treatment is needed. Several authors report colorimetric analysis performed to quantify color modification, microscopy investigations (including SEM) to observe the structural modification that occurred on the paper surface, and pH and alkaline reserve measurements to evaluate the effectiveness of chemical stabilization treatments [14,17,18]. In this work, we compare the spectroscopic markers of paper before and after treatments to evaluate the paper characteristics variation in a non-destructive way.

#### 2.2.1. Hydrogel Cleaning Treatment

Traditional wet restoration methods for librarian and archival artifacts—such as cleaning by immersion—present lots of limitations that are primarily due to the uncontrolled release of water onto the paper supports. It was proposed that to control the quantity and speed of water release through the use of supporting substances, i.e., materials of a certain consistency that contain water or reactive solutions or solvents, as gelling agents, minimize the impact on the paper supports during cleaning treatments. Several gel formulations are currently proposed with different properties and advantages [19,20].

By applying the gel to the paper, the water spreads over its surface, solubilizing the degradation products and contaminants that may be present on it (starch, gum Arabic, animal glues, gelatin). The concentrated solution of the degradation products migrates following the concentration gradient from the paper toward the gel where it is reabsorbed. When the gel is removed, there is a removal of contaminants that are on and within the paper surface.

**Figure 4:**
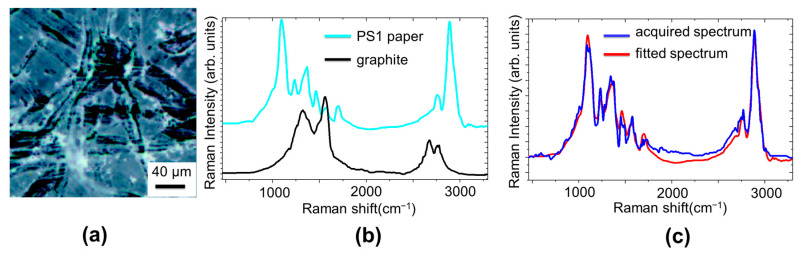
(**a**) Optical image of the paper (PS1) area analyzed before hydrogel treatment. (**b**) Raman spectra of PS1 paper (cyan line) and graphite contaminant (black line) used as reference spectra for loadings to attribute the scores of the linear combination in the CLS procedure. (**c**) Graph of linear combination spectrum obtained by CLS procedure (fitted spectrum in red line) compared with the measured spectrum (blue line).

To study the capability of removing a stain with hydrogel, we used the PS1 paper contaminated with graphite of pencil and a commercially available hydrogel (Nanorestore gel^®^). In Figure 4, it is reported the microscope optical image of the paper area that we analyzed before and after hydrogel treatment.

To describe the cleaning process, it is more convenient to use a different coding of Raman maps, based on the Classical Least Square (CLS) fitting. In this case, the measured Raman spectra, acquired from each point of the prefixed grid, are considered a combination of suitable reference spectra that are used as loadings to attribute the scores of the linear combination. In the present case, the loadings, reported in Figure 4b, are the Raman spectra of PS1 paper (cyan line in Figure 3b) and graphite contaminant (black line in Figure 3b). Their combination, with the appropriate coefficients, reproduces accurately the measured spectrum, as shown in Figure 4c.

**Figure 5:**
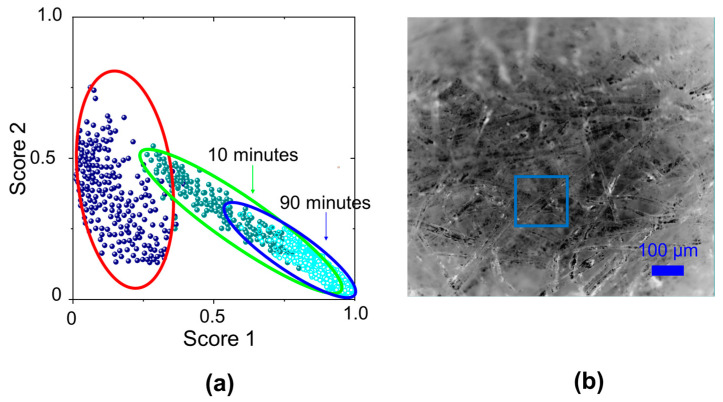
(**a**) Map of the linear combination coefficients of CLS fitting procedure obtained before (points in red ellipse) and after hydrogel treatment for different application times (points in green and blue ellipses respectively) of the PS1 paper sample; x-axis reports the score 1 referred to paper spectrum component and y-axis reports the score 2 referred to graphite spectrum component. (**b**) Optical image of the paper (PS1) sample, the blue square box indicates the area analyzed after 90 min hydrogel treatment.

Following this procedure for each point of the grid, we built the plot in Figure 5a, by reporting for each Raman spectrum the coefficients of linear combination in an x, y plane, with on the x-axis the score of paper spectrum component and on y-axis the score for graphite spectrum component. This results in points with a large score value in y and a small score value in x for the paper contaminated with graphite particles (red ellipse). After the application of hydrogel for 10 and 90 min, a new Raman map is acquired and a new set of core values is calculated (green and blue ellipses). The ellipse that encloses the score points moved to the right lower corner of the x, y plane, indicating a good removal of graphitic particles due to the hydrogel cleaning action, as shown in Figure 5b where the blue square box indicates the sample region that we analyzed.

**Figure 6:**
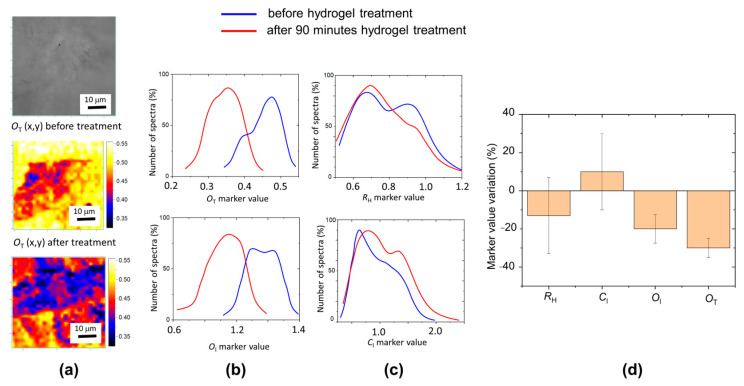
(**a**) Upper, optical image of XIX century paper (Brehm 1896) area before hydrogel treatment. Raman map of *O*_T_ marker before and after 90 min of cleaning procedure. (**b**), (**c**) *R*_H_, *C*_I_, *O*_I_, and *O*_T_ marker distributions before (blue line) and after (red line) hydrogel treatment (t = 90 min). (**d**) Variation after 90 min hydrogel treatment of marker distribution mean values with the corresponding distribution widths.

To evaluate the effectiveness of the removal of oxidation products, we applied Nanorestore gel^®^ to an old paper sample of the XIX century (Brehm, Enciclopedia Vita degli animali, vol VI, Torino 1896). After each cleaning step (10 and 90 min) we acquired a Raman map in the same sample region, obtaining the aging marker distribution values as shown in Figure 6. There is a clear decrease in oxidation markers, in agreement with the increase in pH reported in the literature for similar treatments [19]. However, there is a change also in the distribution of *R*_H_ and *C*_I_ values. The variation of the marker distribution mean values are reported in Figure 6 along with the corresponding distribution width.

After hydrogel treatment, the observed decrease of the *R*_H_ marker and increase in the *C*_I_ marker of about 10% is within the distribution width, differently from the decrease in *O*_I_ and *O*_T_ oxidation markers (−20% and −30%, respectively). Overall, our measurements indicate that hydrogel cleaning is an effective method to remove contaminant and oxidation products with a small variation of paper characteristics as *R*_H_ and *C*_I_ marker (polymerization and crystallinity degree) that is comprised of the variability of these indexes in the macroscopic sample.

#### 2.2.2. Application of Raman Spectroscopy with Mapping to EUV Paper Irradiation Treatment Evaluation

Different cleaning methods are available for paper depending on the particular contaminant. Fungal contamination plays a considerable role in the deterioration of book heritage, catalyzing the decomposition of cellulose and of some related polysaccharides [21]. In this respect, laser cleaning technology has some advantages over conventional cleaning methods, being a contactless and chemical-free technique [22]. To study the best operating conditions for cleaning the paper surface, in 1997 an EUREKA-EUROCARE project entitled “Laser Cleaning of Paper and Parchment” (“LACLEPA”; European Union Project Number 1681) was launched. In this framework, several experiments were performed on new and ancient paper and parchment [23,24,25,26]. Laser cleaning experiments focused on the removal of fungi and other stains from paper surfaces using near UV, visible, and IR lasers, were reported [24,25].

The irradiation of cellulose with UV radiation resulted in photo-oxidative degradation of the paper surface with an increase of carbonyl and carboxyl content and a decrease in polymerization degree. Differently, after the IR irradiation at 1064 nm, the polymerization degree of paper increases due to the inter- and intra-molecular cross-links of ether origin, which was manifested by an increase in the polymerization degree of the treated cellulose [23,24,25,26]. The irradiation in the visible range, when applied to paper contaminated with carbonaceous pollutants resulted to be very effective in the removal of the carbon dirt. However, a clear discoloration of the sample occurs, accompanied by a yellowing effect [24,25,26].

Although it was demonstrated that radiation of 308 nm or less is not suitable for the cleaning of cellulose-based materials, as they may have a detrimental effect on the paper surface, an excimer laser at 248 nm was used to sterilize fungal-contaminated paper [22]. The laser removal of fungi was successful, and suitable parameters that do not alter the paper structure were found, but the plasma cloud produced by laser on the paper surface may contain viable organisms, and precautions should be taken to avoid contamination of the workroom. For this reason, it was suggested that the cleaning process should be performed in an enclosed space, with a vacuum purge [22].

As an alternative method for paper surface cleaning from contamination, we studied the effect of an Extreme Ultra Violet (EUV) irradiation at 10–18 nm in a vacuum, by using a discharge-produced plasma (DPP) EUV source [27,28]. In the case of UV photons, electrons can be excited from the valence band of polymer materials, the excited states can relax through radiative or non-radiative processes and one of the possible radiation-less channels corresponds to the bond breaking of the polymer chain. Differently, a single EUV photon carries a much higher energy, sufficient to ionize any atom or molecule, with the production of photoelectrons that, in turn, have sufficient energy (up to 100 eV) for further excitation or ionization of the polymer molecules. Non-radiative de-excitation processes, including bond breaking, are predominant [29]. Some volatile fractions can be released from the irradiated layer. The EUV smooth ablation is more intense on the amorphous part of the polymer surface. However, the bulk properties of the polymer are preserved, due to a strong EUV absorption in a very thin near-surface layer, of the order of 100 nm.

**Figure 7:**
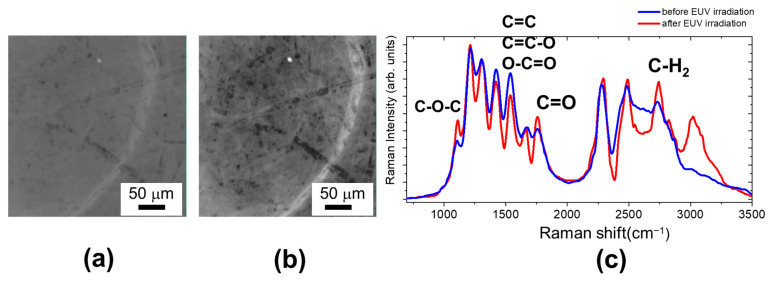
Optical images of analyzed area of 1893 sample paper before (**a**) and after (**b**) EUV irradiation at a fluence of 200 mJ/cm^2^. (**c**) Raman spectra of 1893 sample paper before (blue line) and after EUV irradiation (red line).

In Figure 7, we report the optical image of the analyzed area of a sample paper taken from a XIX century book (O. Tedone, Note sul moto di un fluido, Pisa, 1893), with the Raman spectra before and after EUV irradiation at 200 mJ/cm^2^. By comparing the optical images, after irradiation, the web cellulose structure is more evident, probably due to a more intense ablation of the disordered and amorphous part of the paper surface.

**Figure 8:**
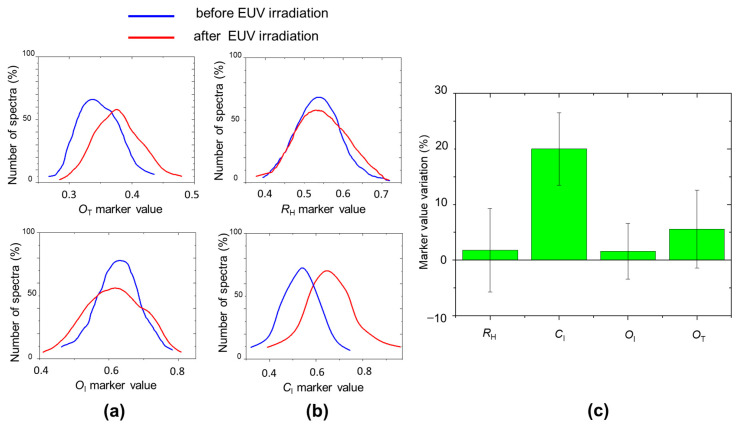
(**a**,**b**) *R*_H_, *C*_I_, *O*_I_, *O*_T_ marker distributions before (blue line) and after (red line) EUV irradiation at a fluence of 200 mJ/cm^2^; (**c**) Variation, after EUV irradiation, of marker distribution mean values with the corresponding distribution widths.

The most evident effects of EUV exposure are the highlighting of the C-O-C band and the sharpening of the CH_2_ peak. The intensity peak increase indicates the formation of new C-O-C groups, suggesting the occurrence of reactions forming covalent cross-links, by ether bonds [24,25]. It is worth noticing that this is not accompanied by an appreciable increase in *R*_H_ parameter if we take into account all the Raman spectra acquired following the grid of 25 × 25 points (see Figure 8c). The increase in C=O peak indicates that there is oxidation with carbonyl formation, although the variation of the *O*_I_ index also is not so evident considering all the analyzed areas.

The significant increase of the CH_2_ stretching peak and the appearance of a band above 3000 cm^−1^ in the OH stretching region indicates structural changes in the cellulose chain arrangement, which affects the degree of crystallinity, evaluated through the *C*_I_ marker increase of 20%, well above the intrinsic variation of this marker, as shown in Figure 8c. The crystallinity index increase can be related both to the formation of additional links that helps to arrange the fiber in a more ordered way and to the fact that the EUV irradiation preferentially ablates more superficial aged layers and amorphous part.

## 3. Discussion

We applied a diagnostic protocol based on markers derived from Raman spectra features, related to polymerization, oxidation, and crystallinity degrees of cellulose fibers. The coupling of a Raman spectrometer to an optical microscope represents a powerful tool to characterize papers allowing one to obtain 2D Raman spectroscopic marker maps at different fields of view by using different magnifications, with a characterization going from the single cellulose fiber to larger areas of the paper. The 2D marker maps can be overlapped to optical image visualizing a spatial resolved compositional/degradation imaging on morphological properties of the paper samples. Moreover, the Raman map measurements in the 2D scanning detection collect a large number of Raman spectra that allow one to obtain marker distributions on prefixed areas for the characterization of modern papers, for evaluation of the aging process, and for cleaning procedures of old books. Therefore, this protocol allows us to appreciate the variation of the paper structure parameter comparing this variation with the variability of the same parameter due to the inhomogeneity of the paper system. In this way, the real variation of paper parameters due to the impact of treatment is revealed.

In our preliminary inspection, the hydrogel treatment was suitable for removing carbonaceous dirty and oxidation products. This method is not safe for the removal of other types of stains, for example, fungi. The EUV irradiation can ablate stains, amorphous, and denaturation products of cellulose, producing an increase in the *C*_I_ index.

However, in our work, we evaluated the short-term effect of cleaning treatment. These measurements should be repeated after a cycle of accelerated aging.

## 4. Materials and Methods

We studied several paper samples: old papers of the XIX century (O. Tedone, Note sul moto di un fluido, Pisa, 1893; Brehm, Vita degli Animali, vol. 6, Torino 1896) from books belonging to author library heritage and modern papers (Whatman^®^ filter lab, Funori (Japan), Antaimoro (Madagascar) with very different compositional and morphological characteristics.

Whatman^®^ cellulose filter papers are manufactured from cotton fibers that are treated to achieve an α-cellulose content > 98%. Funori is a polysaccharide (funoran) obtained from the mucilage of three seaweed species living in the sea waters of Japan, Korea, South China, and North America, i.e., Gloiopeltis Furcata, Gloiopeltis Complanata, and Gloiopeltis Tenax. Antaimoro paper is produced from the bark of the avoha, a Moraceae shrub.

We analyzed the papers by collecting Raman spectra in operando mode, before and after EUV irradiation/hydrogel treatments. Commercial hydrogel material Nanorestore gel^®^ was used for the treatment of old paper.

To irradiate papers, we used the Xe-fed laboratory-scale EUV DPP source operating at the ENEA Frascati research center. The DPP working principle can be summarized as follows: low pressure (0.5−1.0 mbar) Xe gas, filling a 3 mm in inner diameter and 12 mm in length alumina tube, is pre-ionized by a first low current slow discharge (20−30 A, 10−20 μs); after a delay of few microseconds with respect to the start of the pre-ionizing discharge, a low-inductance 50 nF capacitor, charged to 25 kV, produces a second (main) high current fast discharge (13 kA peak current, 240 ns half period duration) in the pre-ionized Xe gas. The resulting magnetic field (>1 T at the alumina tube inner radius) pinches the plasma toward the tube axis; then, the plasma resistance rises, and the plasma temperature can increase up to 30−40 eV. The hot plasma emits EUV radiation before relaxing and cooling. The DPP emits more than 30 mJ/sr/shot in the 10−18 nm wavelength EUV band (selected by a 150 nm Zr filter) at a 10 Hz repetition rate. The duration of each EUV pulse is about 100 ns fwhm; the optical EUV source transverse size is less than 300 μm. The papers were irradiated with EUV fluence of 200 mJ/cm^2^.

2D Raman maps were performed by a Raman spectrometer combined with a confocal microscope (Horiba XploRA Plus, Palaiseau, France) equipped with 532 and 638 nm wavelength lasers. When a Raman spectrometer is combined with a confocal optical microscope it is possible to acquire the information of a complete Raman spectrum in each point of 2D maps by scanning the sample in XY directions. This confocal micro-spectrometer can operate also in luminescence mode. The Raman signals were collected through a microscope equipped with 5×, 10×, 50×, and 100× objectives. Laser power can be attenuated by neutral density filters. After preliminary studies, suitable conditions for both laser power and acquisition times were selected to obtain a good S/N ratio and safe conditions for the paper. More precisely, a 638 nm laser was used to excite the Raman signal, with a power of 15 mW. Raman spectra were recorded at every point of the selected sample area following a prefixed grid defining a 2D map and building a 2D array of spectra, in the 100–3500 cm^−1^ range, with an acquisition time of 1 s for each spectrum. During surface scanning, the focus was automatically maintained by the autofocus procedure of the Horiba spectrometer. The recorded spectra were background subtracted and smoothed, peak area and intensity has been calculated by the software (Labspec 6, version 6.5).

## 5. Conclusions

In the field of book heritage, it is of crucial relevance the study and the development of non-destructive diagnostic protocols for characterizing the state of conservation of the paper and their degradation processes and for evaluating, with in operando procedure, the effects of non-invasive treatments for cleaning/disinfecting paper surface to slow down degradation processes.

In this work, we applied a previously developed diagnostic protocol to characterize modern papers with very different compositions and properties, to characterize the degradation state of old papers, and to evaluate in operando mode (before and after treatment) two different cleaning processes based on the application of hydrogel material and on the EUV irradiation. The obtained results demonstrate that this protocol is a very appropriate diagnostic method for studying the effects of these treatments as non-invasive restoring procedures of old paper and to characterize the unwanted changes that the treatments should produce in the structure of the paper, indicating that it is a powerful tool to develop new cleaning procedures.

## Data Availability

The data presented in this study are available on request from the corresponding author. The data are not publicly available to ensure that the data are not mishandled.
